# Newer kid on the block

**DOI:** 10.1016/j.igie.2025.03.001

**Published:** 2025-03-22

**Authors:** Scott VanderMeer, Linda S. Lee

**Affiliations:** 1 2855 Boardwalk Drive | Ann Arbor, MI 48104 MTEndoscopy.com; 2 Brigham and Women's Hospital, Endoscopy Center, 75 Francis St, Boston, MA 02115

## Editor’s Introduction

Although gastroenterologists cannot perform endoscopy without endoscopes, they also cannot perform diagnostics (beyond endoscopic visualization) and therapeutics without endoscopic accessories. The importance of accessories cannot be overemphasized as they have revolutionized endoscopic retrograde cholangiopancreatography (ERCP), endoscopic ultrasound (EUS), and third-space endoscopy. Furthermore, subtle features on accessories may have tremendous impact on the outcome of the procedure, for example, hemostatic clips, in that those with shorter tails allow use over the elevator of a duodenoscope. Although several companies dominate the U.S. market for endoscopic accessories, a newer entrant has made waves in recent years. I am delighted to discuss the evolution of Micro-Tech (Ann Arbor, Mich) in the United States with its President, Scott VanderMeer. Scott has extensive experience commercializing new technologies in the United States and over 20 years of healthcare experience in the medical device field. Before joining Micro-Tech, Scott worked with other innovation-led companies, including Verathon Medical (Sarasota, Fla, USA) and Clarius Imaging (Vancouver, BC, Canada). These companies have achieved market leadership positions in their respective categories, highly in part because of their extreme commitment to innovation. Scott joined Micro-Tech in 2022 as Vice President of Sales and now leads comprehensive business efforts as President of the Americas.

**Linda Lee (L.L.): Thank you so much for taking the time to discuss Micro-Tech with me. Can you first summarize Micro-Tech's history?**

**Scott VanderMeer (S.V.M.)**: Micro-Tech began in the year 2000 in Nanjing, China, with our first China Food and Drug Administration approval for esophageal and biliary stents. By 2003, we became China’s first manufacturer of single-use biopsy forceps, earned Conformite Europeenne approval for stents and forceps in Europe, and launched Micro-Tech Europe in 2007. Like many Chinese MedTech companies then, we lacked global brand recognition. That changed in 2015 when leadership drove a strategic entry into the U.S. market. We went public on the Shanghai Stock Exchange’s Science and Technology Innovation Board in 2019, further fueling our growth. Today, Micro-Tech is a leading global supplier for gastrointestinal (GI) endoscopy, with over 2000 employees serving healthcare providers in 90+ countries.

**L.L.: Why did Micro-Tech enter the U.S. market in 2015?**

**S.V.M.:** The United States represents about one-third of the global GI endoscopy market, but it has long been dominated by a few major players. We saw an opportunity to offer U.S. physicians and patients something different: “value-based innovation.” Our goal was simple—improve clinical benefits with affordable products, free of restrictive contracts. U.S. physicians and business partners were seeking change, so Micro-Tech entered the U.S. market with a fresh approach based on the perceived market opportunity.

**L.L.: How did Micro-Tech gain market share in such a competitive space?**

**S.V.M.:** Breaking into the U.S. market is never easy. Hospitals were tied to group purchasing organization contracts, and our brand recognition was low. Instead of fighting uphill, the team targeted ambulatory surgery centers, an overlooked but growing market segment. Ambulatory surgery centers are price-sensitive and value-efficient, so Micro-Tech offered a hassle-free experience: no contracts, no minimums, just quality products at a fair price. The team offered a fair price by embracing automation, robotics, and mass production.

**Figure 1:**
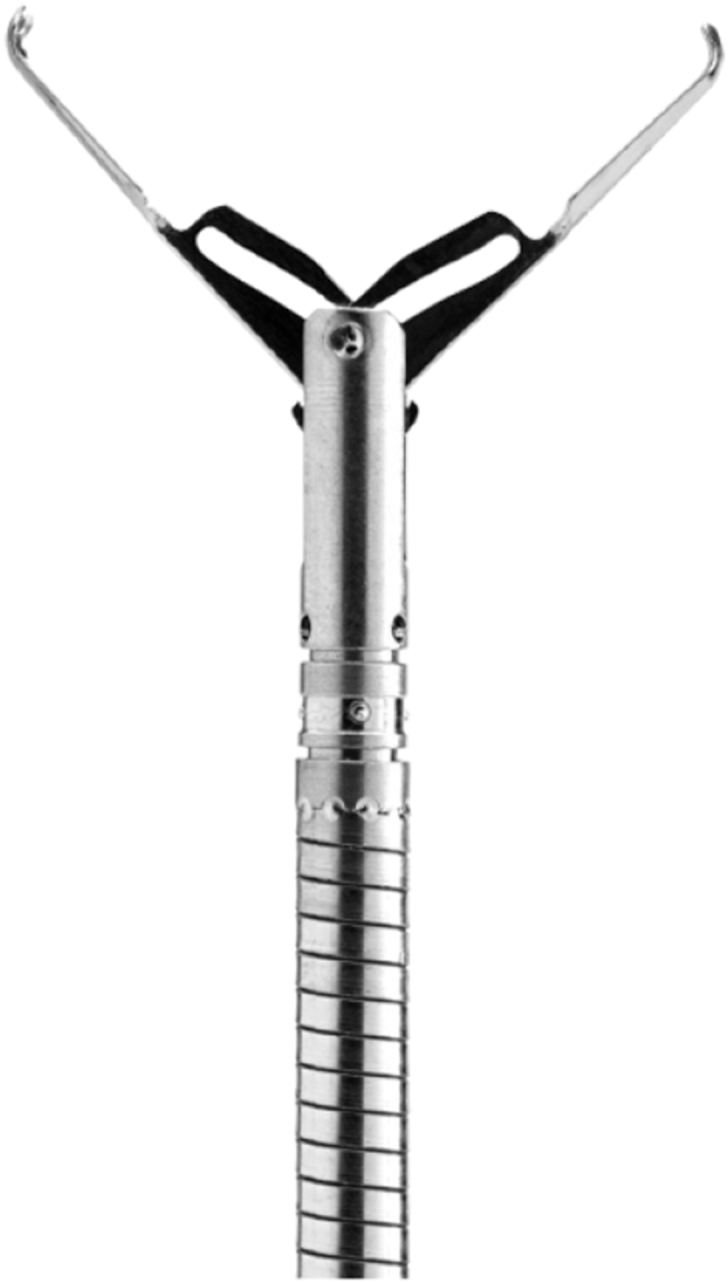
Sureclip.

At first, the team relied on competitive pricing, knowing customers would quickly learn Micro-Tech delivered more than just affordability—our products also deliver exceptional quality. The SureClip hemostasis product family, launched in 2016, became Micro-Tech’s breakout moment (Fig. 1). The proprietary clip design provides reliable deployment, may improve retention, and offers a choice of jaw sizes.

Physicians loved its performance, and SureClip earned us a reputation as the “clip company.” This opened doors to hospitals, where clips are widely used. From there, the engineering and marketing teams further expanded our portfolio, building a full line of diagnostic and therapeutic tools to meet the needs of both ambulatory surgery centers and hospitals.

Our customer-first approach awoke the market. Physicians appreciated the flexibility and freedom we offered, and they were excited to finally have a choice beyond the few big players. Once they evaluated our products, any concerns about quality disappeared. They saw firsthand our commitment to performance and unquestionable reliability. This remains a high priority for us today.

**L.L.: Pivoting to discuss another of your products, why did Micro-Tech focus on cholangioscopy and what challenges did you face developing the eyeMAX cholangioscope?**

**Figure 2:**
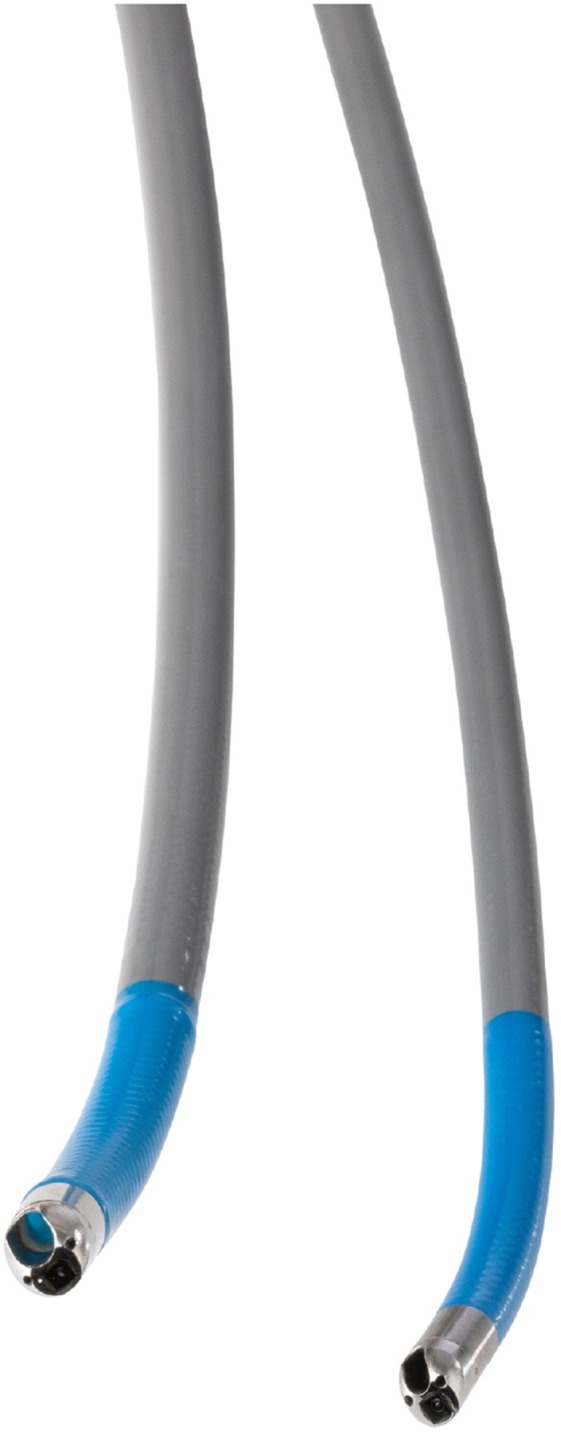
eyeMAX cholangioscope.

**S.V.M.:** Micro-Tech employs over 200 world-class engineers and a support team that extends far beyond that. Cholangioscopy is a growing field, and Micro-Tech prioritized scope innovation because of the lack of innovation in the market. The biggest challenge in developing our cholangioscope was balancing optical performance, illumination, a large instrument working channel, and mechanical precision on such a small endoscopic tip while keeping manufacturing efficient and cost-effective. Our engineers worked closely with physicians for years, iterating until we found the perfect balance. The result is a cholangioscope that advances the standard of care (Fig. 2).

**L.L.: How long did FDA (U.S. Food and Drug Administration) clearance take for eyeMAX and what were the key steps?**

**Figure 3:**
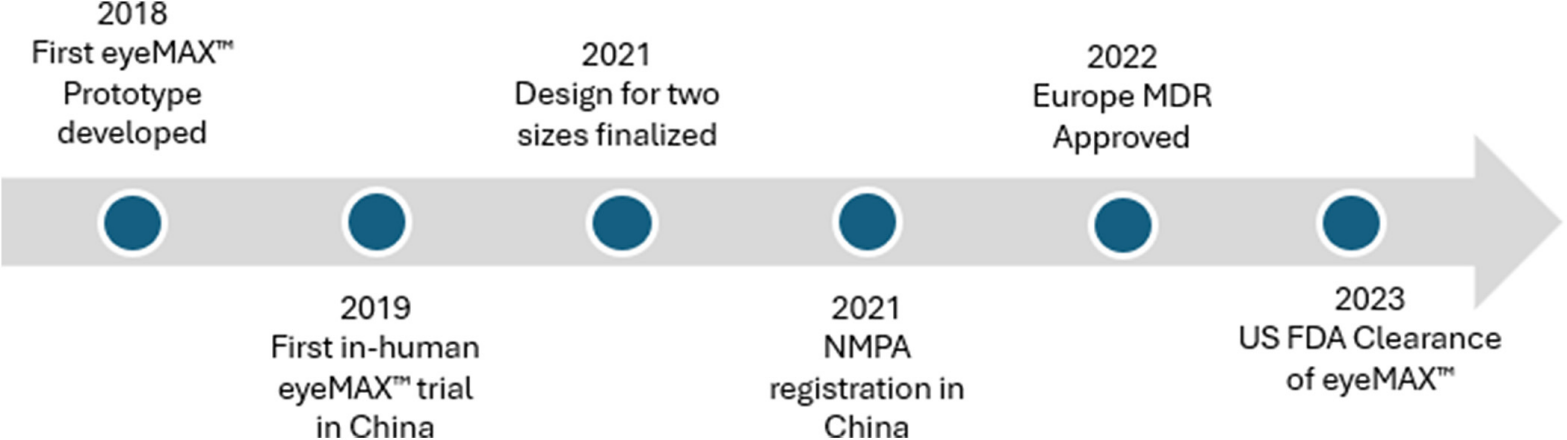
Timeline of key events in development of eyeMAX. *NMPA*, National Medical Products Administration; *MDR*, medical device registration; *FDA*, U.S. Food and Drug Administration.

**S.V.M.:** The FDA clearance process took about 10 months (Fig. 3). We worked closely with the FDA, answering regulatory and technical questions while providing robust preclinical and clinical data. Micro-Tech’s eyeMAX had already demonstrated success in Asia and Europe, so we leveraged that experience to prove its safety and efficacy.

Key advancements in eyeMAX include exceptional image quality, 2 scope sizes, and a larger operative channel that accommodates a broader range of instruments, allowing more diagnostic and therapeutic options to be delivered through the scope. Each feature required rigorous testing and validation to meet FDA standards. The result is a device that gives physicians exceptional visualization, high procedural flexibility, and enhanced patient treatment options.

**L.L.: How does Micro-Tech approach global product launches?**

**S.V.M.:** Every product launch follows a thoughtful, region-specific strategy. We prioritize key markets based on clinical demand, regulatory pathways, and our ability to support training and education. With eyeMAX, this approach ensured rapid adoption in major regions worldwide. We began with a product launch in Asia as we are headquartered in China and have more resources to move products through the regulatory process faster in Asia and Europe.

**L.L.: What’s in the pipeline for endoscopy?**

**S.V.M.:** We continue to focus on ERCP, endoscopic mucosal resection (EMR), third-space, and EUS procedures—areas with significant unmet clinical needs. Micro-Tech’s speed is unmatched: We can go from physician concept to prototype in months. We also utilize our onsite preclinical testing facility and state of the art equipment to evaluate the prototypes with physicians, greatly shortening the development cycle. It’s exciting to see solutions come to life so quickly and to help solve complex healthcare problems.

**Figure 4:**
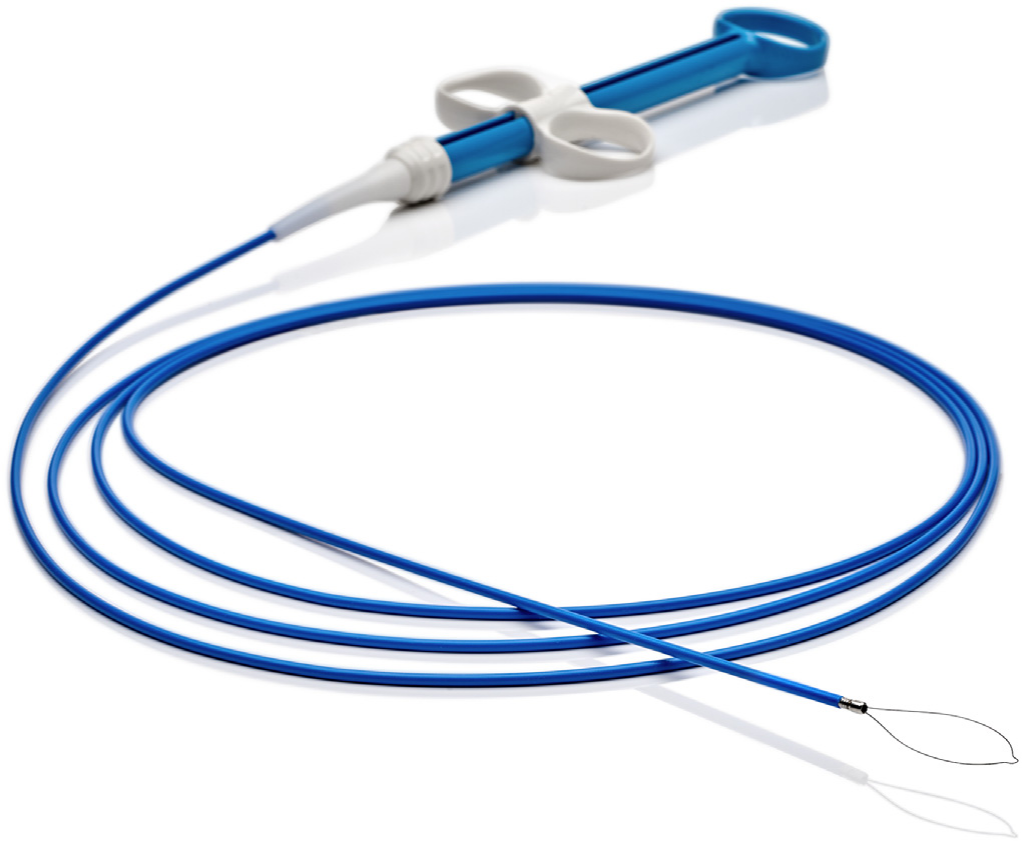
LesionHunter rotatable cold snare.

**Figure 5:**
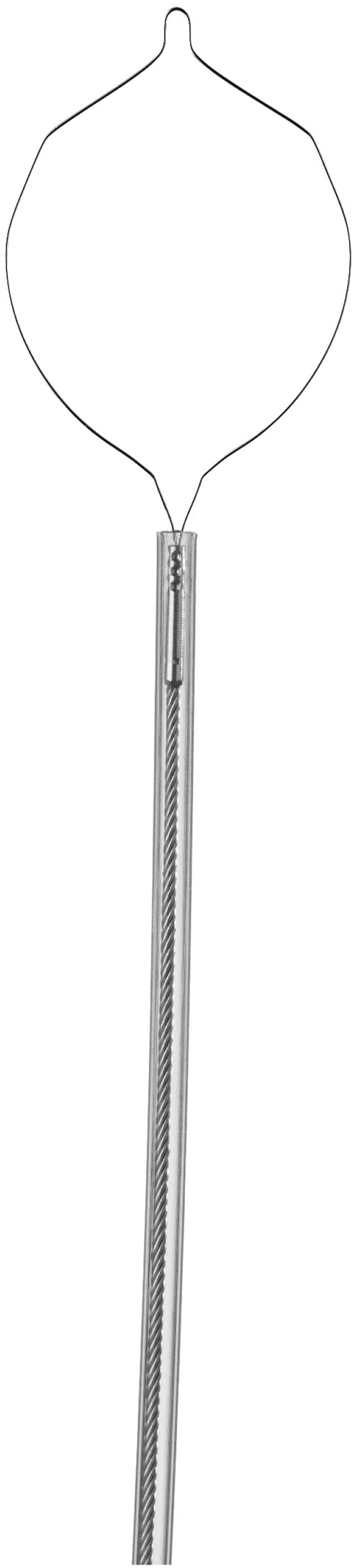
EdgeHog rotatable hot snare.

Two examples are our recent launches of LesionHunter (Fig. 4), a rotatable nitinol cold snare for efficient tissue resection, and EdgeHog (Fig. 5), a rotatable hot snare designed for clean, precise cuts during advanced EMR procedures. Both tools reflect our commitment to physician feedback and innovation.

In the ERCP space, we continue to expand the eyeMAX platform with new accessories to give physicians even more flexibility. We also have significant innovations in third-space endoscopy and EUS on the horizon. Collaborating with physicians keeps our pipeline strong, and we’re excited about what’s next.

**L.L.: What’s the future vision for Micro-Tech?**

**S.V.M.:** Micro-Tech began 24 years ago with a clear mission: deliver high-quality, cost-effective endoscopy products while being friendly to work with. That mission hasn’t changed. What’s different today is our scale—we’re now recognized as a global innovator.

Our vision is to keep pushing boundaries. By collaborating with physicians now worldwide, we aim to develop game-changing technology that transforms endoscopy and improves patient outcomes. The future is bright, and we’re just getting started.

## Editor’s Conclusion

Simon Sinek wrote, “Competition is the fuel that ignites innovation.”^1^ This has certainly proven true with Micro-Tech entering the U.S. market using an approach focused on quality and value. Its ability to innovate rapidly has led to the second single-use cholangioscope on the market in over 15 years since the introduction of Boston Scientific’s single-use, single-operator cholangioscope system. Gastroenterologists are always seeking finer tools to provide better, more efficient, durable care to our patients. Our symbiotic partnership with industry remains critical in continuing to move our field forward.

## Disclosure

The following authors disclosed financial relationships: S. VanderMeer: Full-time employee of Micro-Tech. L. S. Lee: Consultant for Boston Scientific, Fujifilm Healthcare Americas, Fractyl, and Cook Medical.
